# Operationalising a model framework for consumer and community participation in health and medical research

**DOI:** 10.1186/1743-8462-4-13

**Published:** 2007-06-26

**Authors:** Carla Saunders, Sally Crossing, Afaf Girgis, Phyllis Butow, Andrew Penman

**Affiliations:** 1 School of Medicine and Public Health, The University of Newcastle, Wallsend NSW, 2287, Australia; 2 The Cancer Council NSW, Woolloomooloo NSW, 2011, Australia; 3 Cancer Voices NSW, Greenwich NSW, 2065, Australia; 4 Centre for Health Research & Psycho-oncology (CHeRP), The Cancer Council NSW, University of Newcastle & Hunter Medical Research Institute, Newcastle NSW, 2287, Australia; 5 Medical Psychology Research Unit, University of Sydney, Camperdown NSW, 2006, Australia

## Abstract

The Consumers' Health Forum of Australia and the National Health and Medical Research Council has recently developed a *Model Framework for Consumer and Community Participation in Health and Medical Research *in order to better align health and medical research with community need, and improve the impact of research. Model frameworks may have little impact on what goes on in practice unless relevant organisations actively make use of them. Philanthropic and government bodies have reported involving consumers in more meaningful or collaborative ways of late. This paper describes how a large charity organisation, which funds a significant proportion of Australian cancer research, operationalised the model framework using a unique approach demonstrating that it is both possible and reasonable for research to be considerate of public values.

## Background

The National Health and Medical Research Council (NHMRC) is Australia's national independent agency responsible for the overall development of research strategy and the allocation of the vast majority of health and medical research funding [1]. Recently the NHMRC announced that it would make changes to its funding processes in order to better align health and medical research with community need, and improve the impact of research [2]. A commitment such as this signifies an important culture shift toward greater public involvement in health research. This shift is brought about by a range of factors including demands from an increasingly educated and aging population in growing need of health care, and rapid technological advances in diagnostic, clinical practice and treatment options[3].

Actively seeking the views of consumers regarding health research is now recognised as an opportunity to access valuable untapped knowledge, and a means of improving the relevance and translation of research into practice and of making research organisations more accountable [4,5]. Consumer perspectives trigger consideration for the wider environmental, psychosocial, and behavioural contexts of people's lives [6]. Further reported benefits include a greater acceptance and uptake of research findings in the community, and more efficient use of research resources [7,8].

Approaches to genuine consumer participation in health research are evolving. Past approaches were based on a false separation of expert from lay person with corresponding activity tending to place the parties in active and reactive roles. Consumers were traditionally involved in symbolic or passive ways, such as sitting on research committees, as research subjects, or as fundraisers for research dollars [9]. Recently, philanthropic and government bodies have both reported involving consumers in more meaningful or collaborative ways including recognising research opportunities through participation in research agenda setting meetings and workshops, to identifying research needs, evaluating research hypotheses, communicating results and supporting the incorporation of appropriate research findings into practice [9-11]. Often, more than one participation strategy is reported [9].

Consumers face particular challenges when they become involved in health research. Some of the barriers experienced include lack of confidence in their decision-making ability in relation to research and a lack of understanding and education about research and research governance. Neglecting to involve consumers in research has previously led to adverse patient outcomes either as a direct result of poorly designed or implemented research or as a result of absent or poor communication of research outcomes [12,13]. Goodare and Chalmers provide convincing arguments for improved levels of consumer involvement through descriptions of 'real life' consequences of overlooking consumers in research processes [12,13].

### A model participation framework

In Australia the importance of consumer participation in research has been recognised in a number of reports and initiatives [14-16]. In partnership with Australia's peak independent organisation representing consumers on national health care issues, the Consumers' Health Forum of Australia (CHF), the NHMRC has recently developed a model framework for Consumer and Community Participation in Health and Medical Research [17]. The existence of a model framework in any field recognises the importance of that field, and is often evidence of seminal thinking in itself. At a minimum, the framework is intended as a resource to provide advice and practical information to support and promote consumer participation in research in Australia. To be helpful, a framework generally has to be relevant to practice and adaptable to local structures, environments and needs. The application of any model framework is subject to operational realities and constraints. In addition, frameworks may make little impact on what goes on in practice unless relevant organisations actively make use of them. A model framework is by its very nature an evolving entity that can be extended and improved over time.

This paper describes how a large charity organisation, which funds a significant proportion of Australian cancer research, operationalised the model framework using a unique approach.

### The Cancer Council NSW Consumer Involvement in Research Project

Influencing the direction of research into the causes, prevention, optimal treatments and support for cancer sufferers is a major goal of a number of Australian cancer consumer advocacy groups who believe that consumers can add significant value to research through their active participation [18,19]. The Cancer Council NSW (CCNSW) believes it is very important that consumers and the wider public value its research, which is principally financed through charitable donations. Consumers have always been involved, to varying degrees, across a range of CCNSW research processes, however it was felt that important opportunities existed for improvements in this area. Collaboration between the peak NSW cancer advocacy organisation, Cancer Voices NSW (CVN) and the Cancer Council NSW, led to an approach that would allow consumers genuine input into the allocation of funding for CCNSW research including the establishment of a dedicated consumer review panel and specific consumer review criteria.

The specific aims of the *Consumer Involvement in Research Project *were to:

• Assess consumers', non-commercial financial donors' and the public's views on what they value as important in deciding what research should be funded.

• Develop a set of criteria suitable for use by cancer consumers and members of the public to rate research applications submitted to CCNSW for funding.

• Identify the type of training that may be required to assist consumers to participate in the research grant review process.

• Establish a consumer panel whose trained members would effectively review, apply specific criteria and give a ranking to CCNSW research grant applications.

• Identify suitable research governance arrangements that formally incorporate public values in research funding decisions.

### Aligning the Consumer Involvement in Research Project with the model framework

The CCNSW project will be described in relation to each of the four fundamental components of the NHMRC/CHF model framework. The four components are considered essential for successful consumer involvement in research.

#### Model framework: Key component 1

*Both senior leadership and operational capacity will underpin success in developing consumer and community participation in research. Whilst all researchers have a part to play in developing consumer participation, it is helpful to have a designated person who is responsible for ensuring progress at an organisational level and who can facilitate the attempts of researchers and consumers to work together*.

In mid 2004 the CCNSW Board of Directors, considering a proposal put forward by the Chair of Cancer Voices NSW and recognising the value of consumer participation in the direction and process of research, affirmed the Cancer Council's commitment to this issue by endorsing an investigation into a workable organisational solution, including any associated structural and process changes, to involve consumers in the research grant review process. A senior staff member, who was assigned initial responsibility for development of the concept, saw the initiative through to completion and continues to provide advice regarding changes and enhancements to the consumer review process. For the most part, this staff member assumed day-to-day responsibility for the progression of the project, made sure all decisions considered the needs of relevant parties who would be affected by required changes and was to all intents and purposes confined to this one project. According to the complexity theory [20], placing a large number of people on a single project creates more interplay than progress.

Supporting structures such as a project advisory committee and research team, both of which included informed consumer representatives, were established to guide the necessary changes to support the consumer review of research grant applications including advising the development of necessary tools, guidelines, education materials and evaluation and reporting procedures. A separate research team led an investigation to identify the values deemed by consumers and the public to be important in guiding lay people in objectively judging the value and merit of different research. The prevailing public and consumer needs were to become the 'consumer review' criteria within a formal appraisal tool to guide lay people in objectively judging the value of different research.

#### Model framework: Key component 2

*Building consumer and community participation into the structures of research funding bodies, organisations and teams will strengthen and support its implementation*.

Existing administrative and cultural characteristics were key considerations in the development and fit of consumer involvement in the CCNSW review process. In the past, CCNSW research funding decisions were based solely on scientific merit criteria that fundamentally estimate the potential success of research through an assessment of scientific quality and the ability of the investigator to conduct the research. A number of national specialty committees covering a range of research disciplines assigned numeric ratings to the relevant funding applications. Consumers were not involved in the review of CCNSW research grant applications against scientific merit criteria. As public accountability inevitably requires research funds to be properly administered to those with the highest likelihood of success, it was necessary to maintain the integrity of the scientific review process.

It was agreed by all parties that the review of research grants would involve a two-stage assessment process with a difference existing in the membership of the two layers of committee structures. The two tiered structure, which allows the CCNSW to strike a balance between funding research of significant scientific merit, and research judged especially relevant by consumers is provided in Table 1. It is a workable structure for CCNSW because the demand for research funding consistently exceeds the supply of CCNSW funds and choices have to be made, even amongst applications that have significant scientific merit.

**Table 1:** The Cancer Council NSW grant review process

**Step 1: National Health and Medical Research Council (NHMRC) review**
Applicants for research grants must demonstrate that the research has the relevant approval (eg human ethics, animal ethics, bio-safety) from a recognised research ethics committee before a grant is funded. Specialty research committees review research grant applications based on scientific merit criteria and give a rating and ranking to eligible applications which are then forwarded to CCNSW for a further assessment against consumer review criteria.

**Step 2: Consumer review**

A trained Consumer Review Panel assesses the eligible funding applications (pre-selected through the scientific review process) based on consumer review criteria and assigns a separate 'public value' weighting to each. An overall priority ranking for the applications is developed identifying those that best satisfy the consumer review criteria.

**Step 3: Cancer Research Committee recommendations**

The Cancer Council's Cancer Research Committee (CRC) formally reviews funding applications that have been assigned a priority ranking against both scientific merit and consumer review criteria, and makes final funding recommendations to the CCNSW board.
In making their funding recommendations, the CRC gives the rankings from each group equal weighting and assesses any major discrepancies between consumer and scientific review to ensure that the final list of fundable projects is the most appropriate.

Rather than scientists and consumers collectively deciding on funding outcomes based on scientific merit, an independent consumer structure and the identified public value measures would allow consumers the opportunity to weight research applications based on criteria relevant to them. Far from requiring significant organisational change and being inherently complicated, the strength of the selected approach i.e. the creation of a secondary review process, allowed existing structures to remain, while fully supporting consumer choices.

It was acknowledged that researchers, by nature of being human, create and maintain particular norms, values and culture within their area of influence. It was found that an independent consumer structure in which consumers were able to openly contribute to discussions and negotiations amongst consumer peers had a more informal and unrestricted atmosphere. Consumers, rather than feeling they are required to follow the lead and opinions of researchers, now have an objective say in the research ultimately funded by the CCNSW. It also means that there is no expectation that consumers should develop any sophisticated expertise in scientific review criteria such as research design, methodologies or analyses. Their 'expertise' is their ability to provide an informed consumer perspective to the decision-making process. CCNSW commitment to consumer participation in research review was built within the existing review framework while providing an important level of consumer autonomy.

#### Model framework: Key component 3

*Resources are needed to help consumer and community participation to work well*.

The costs and benefits of consumer involvement must be identified. Once the project strategies were agreed, a detailed written plan was developed, including defining the work requirements, establishing a division of responsibilities, and quantifying the types and numbers of resources required, such as staff time requirements. This approach ensured that all project activities and necessary resources were properly defined. The more tangible resources used in the project such as the research contract with our university partner, training manuals, mailouts and other administrative costs etc were easily identified and represented known costs to the organisation. Labour costs were more difficult to quantify and initial assumptions under-estimated the actual outlay in this area.

Organised consumer review of research relies on the commitment of a range of staff right across the organisation. Staff responsibilities include the coordination, training and management of the consumer review panel, and communicating processes, progress and problems to researchers, CCNSW Board and management. Staff also have responsibility to support the practical needs of consumer panel members, serve as resource persons throughout the review process, review training program and manual contents, and consider ways to continually improve the consumer involvement procedures and systems.

The commitment and ongoing support of key consumers, particularly the time and opportunity costs, also need to be factored into an assessment of the resources required to successfully undertake a project of this nature. Formally registering as volunteers of the CCNSW, consumers have been significantly involved over many months in the genesis, conduct and promotion of this initiative. The active, enthusiastic involvement of this group continues unwavering today. While the CCNSW volunteer reimbursement policy provides for full compensation for all out of pocket expenses incurred as a result of volunteering for the CCNSW such as phone calls, motor vehicle expenses, parking, child care etc, volunteers offer their valued services willingly with no expectation of any additional payment.

#### Model framework: Key component 4

*Developing and sustaining consumer and community participation requires changes to structures and attitudes, which take time and commitment*.

It was recognised that effective consumer participation in research requires adequate support, knowledge, skills and resources for both consumers and researchers. A core expectation of consumer participation is that they will wish to participate. The independent consumer panel charged with reviewing research applications was established through a voluntary 'opt in' and formal assessment basis. To minimise the effect of real-world factors such as differing levels of experience and expertise among potential members, applicants were purposefully selected based on a number of aspects. These included whether they had an interest in cancer research, a willingness to familiarise self with research terminology, disciplines and concepts, experience in consumer centred cancer groups and organisations, and the ability to keep up to date with current consumer issues via consumer networks and associations.

There was also a need to develop consumer panels in ways that created an ongoing commitment to organisational goals. This was achieved through the development of formal terms of reference, comprehensive review guidelines and the provision of adequate training and information. To prepare consumers to review research funding applications, all necessary knowledge and skills, topics, tasks, and assessment requirements were identified and developed into a comprehensive annual 2-day training workshop, which is mandatory for all consumer review panel members. The aim of the training, which is based on general adult learning principles, is to ensure that participants:

• Understand CCNSW research mission and its extramural research activities;

• Are familiar with key research concepts and terminology required to participate in the consumer review process;

• Understand the consumer panel member role and responsibilities for participation in the consumer review process.

A training manual with a large variety of printed materials has also been developed to supplement the training and provide a ready reference of essential information to assist consumers in undertaking research review.

In addition to the training requirements, consumer panel members suggested that the review of research grant submissions would be made easier if they were provided a consumer friendly abstract explaining the scientific basis of each application in laymen's terms. This is now a requirement of all CCNSW research project grant applications. Information, including examples of well-developed lay summaries is included in the initial funding application form. Researchers are informed that it is in their best interest to provide adequate detail in the lay summary, as consumers are not expected to have a science or research background or to fully read (or necessarily understand), the original research applications. To their credit the majority of researchers who have applied for CCNSW funds in the past two years have readily responded to the different needs of consumers.

The consumer review criteria, which were designed to assist the consumer panel members to judge research proposals for funding, were developed and established over nine months of research [22] to determine what aspects of research were important to those affected by cancer and to the wider community. Factors that were found to be very important to consumers include the level of positive impact on human lives, whether the research could be applied in the real world and if it would be available to all who could benefit by it, how soon the research was likely to be available in clinical practice, and whether consumers were involved. Training and information in the understanding and use of the review criteria is provided to consumers and researchers. The first year of the consumer review process led to the funding of three out of twelve applications that would not have been previously considered, but which better served the needs of the community.

It has taken over two years of planning, researching, testing and retesting to reach the current level of consumer involvement in research review at the CCNSW.

## Limitations

While the *Consumer Involvement in Research Project *successfully involves consumers in a responsive research funding grant program, it may not be applicable to commissioned research initiatives aimed expressly at meeting identified research and development priorities.

## Conclusion

Relatively unstructured consumer involvement in research can take a great deal of time and effort to examine and sort for valuable insights, and often does not develop into a tangible product that readily identifies both the process and outcomes of consumer involvement. The NHMRC/CHF model framework provides practical advice on structure using four key components that are considered necessary for effective consumer involvement in research. The CCNSW initiative provides a clear example against the four key components of the model for one particular initiative, namely research funding review. We believe this example is a valuable addition to the steadily increasing number of soundly grounded mechanisms for consumer involvement in research.

It has been reported that models for engaging consumers must look ahead and contribute to long term goals, they must aim to deliver desired changes in the real world, be fair and take account all interests and not be afraid of experimentation [21]. The CCNSW consumer review process is a long-term solution to issues where consumers and researchers begin at disparate levels and have different priorities. The project originated in partnership with consumers and has required considerable conceptual, financial, human and infrastructure resources. It places consumer participation within a workable, respectful and lasting structure. Consumer involvement in research funding review at the CCNSW is now a committed, systemic practice underpinned by prevailing public values. It is a model that is eminently translatable by other research funding organisations.
